# Prevalence of Hypothyroidism among Dialysis Patients in Palestine: A Cross-Sectional Study

**DOI:** 10.1155/2020/2683123

**Published:** 2020-05-13

**Authors:** Zaher A. Nazzal, Emad N. Khazneh, Razan A. Rabi, Ahlam A. Hammoudeh, Ahmed F. Ghanem, Mohammed A. Zaidan

**Affiliations:** ^1^Department of Family and Community Medicine, Faculty of Medicine and Health Sciences, An-Najah National University, Nablus, State of Palestine; ^2^Department of Nephrology, An-Najah National University Hospital, Nablus, State of Palestine; ^3^Department of Medicine, Faculty of Medicine and Health Sciences, An-Najah National University, Nablus, State of Palestine; ^4^Department of Radiology, An-Najah National University Hospital, Nablus, State of Palestine; ^5^Department of Endocrinology, Istishari Arab Hospital, Ramallah, State of Palestine

## Abstract

**Introduction:**

The kidney affects the thyroid gland causing various derangements in its function whenever the kidney is impaired, even with a minor imperfection in its job, and this makes dialysis patients more prone to thyroid disorders with subsequent increase in mortality and morbidity. This study aims to assess the prevalence of thyroid disease (hypo- and hyperthyroidism) among dialysis patients and their associated factors.

**Methods:**

This cross-sectional study was conducted in the dialysis unit of An-Najah National University Hospital. 209 dialysis patients (60% were male, 57.6 ± 14.5 years, mean age) meeting our inclusion criteria were tested for thyrotropin (TSH) and free thyroxine (FT4) in addition to routine laboratory tests. *Findings*. The prevalence of hypothyroidism was assessed as 16.3% (95% CI = 11.29% to 21.3%), overt hypothyroidism was 9.1%, and subclinical hypothyroidism was 7.2%. Subclinical hyperthyroidism prevalence was 1%, and no overt hyperthyroidism cases were reported. We observed no significant association between thyroid state and age, gender, duration of dialysis, or weight. *Discussion*. Hypothyroidism (both subclinical and overt type) is commonly seen in dialysis patients, and its symptoms are ordinary complains even in euthyroid dialysis patients, and this warrants screening programs and more studies on the efficacy of thyroid hormone supplements.

## 1. Introduction

The overlap between thyroid disorders and renal diseases had become a major concern in recent decades. Thyroid hormones are necessary for the embryological development and growth of the kidney. On the other side, the kidney has a substantial role in thyroid hormone metabolism, degradation, and elimination [1, 2].

It has been noticed that a high prevalence of thyroid disorders exists among patients of kidney diseases, especially those under dialysis. The exact reason for this association is not well understood, and some hypotheses propose that thyroidal derangement is a result of nonthyroidal illness, in which thyroid hormone impairments are present without a primary gland dysfunction. Moreover, the presence of high iodine stores due to a decrease in renal iodine exertion and subsequent iodine retention, in turn, cause hypo- and hyperthyroidism by Wolff–Chaikoff effect and Jod-Basedow phenomenon, respectively [2, 3]. Furthermore, kidney disorders cause decrease in levels of thyroid hormones due to alteration of their metabolism and catabolism [4, 5], notably Triiodothyronine (T3) level is low in kidney dysfunctions as the conversion of peripheral thyroxine (T4) to T3 is impaired by either malnutrition, metabolic acidosis, or certain medications [2, 6]. Less adopted explanations are dialysis, loss of thyroid-binding proteins and minerals, like selenium [3, 7], and alteration in the hypothalamic-pituitary-thyroid axis. The latter was observed in some studies that found TSH level insignificantly increased in dialysis patients with low T4, thus this indicates an impaired axis [3, 8]. However, the majority of studies observed the contrast, confirming that TSH is a reliable method for the detection of hypothyroidism [5–7, 9, 10].

Regardless of the etiology, it is agreed that end-stage renal disease (ESRD) patients have a higher risk than healthy people for confronting thyroid disorders, with hypothyroidism being the most significant, but the exact prevalence varies between geographic areas. According to US and Asian studies, hypothyroidism prevalence ranges from 13–25% [2]. Yet, most cases of hypothyroidism are missed due to the overlap of its symptoms with ESRD, malnutrition, and uremia symptoms, such as dry skin, constipation, cold intolerance, and fatigue [5, 11, 12].

All this effort to estimate thyroid disease prevalence is because thyroid disorders, both hypo- and hyperthyroidism, can result in higher mortality and morbidity among ESRD patients. About 45% of mortality in dialysis is due to cardiovascular disease, and hypothyroidism is suggested to be a plausible cardiovascular risk factor in this population. Hypothyroidism causes endothelial dysfunction, dyslipidemia, and systolic and diastolic pressure impairment, all of which accelerate atherosclerosis, the main mechanism for cardiovascular events [1, 13]. Additionally, hypothyroidism increases all causes of death in dialysis patients [14]. In a prospective study of 540 hemodialysis patients followed for 2 years, high TSH level was associated with a twofold higher risk of all causes of death compared to euthyroid dialysis patients [10]. Other complications linked to the thyroid disorder include a higher risk for extrathyroidal cancer [3, 15] and less eligibility for kidney transplantation [10, 15]. Furthermore, hypothyroidism in dialysis is associated with lower quality of life, especially in the health-related domains, as they complain of more fatigue, lower level of physical function, and more pain compared to euthyroid dialysis patients [16]. Hence, one could tell that thyroid disorder is a serious condition that may be faced dialysis patients, but the question remains whether thyroid replacement therapy will ameliorate mortality and morbidity, and what is the cu off to treat, as some authors propose that TSH between 5 and 20 IU/ml is considered normal and therapy may be harmful [4].

Our study was designed to investigate the prevalence of hypothyroidism in patients under dialysis in Palestine, where no such prevalence was ever explored, as well as to find the parameters that may be associated with thyroid derangements in this population. The result of this study is expected to aid practitioners in estimating the burden of hypothyroidism on patients under dialysis and to adopt earlier preventive measures, and whether it is huge enough to implant a screening program periodically for early diagnosis and treatment of such condition.

## 2. Materials and Methods

### 2.1. Study Design and Participants

**Figure 1:**
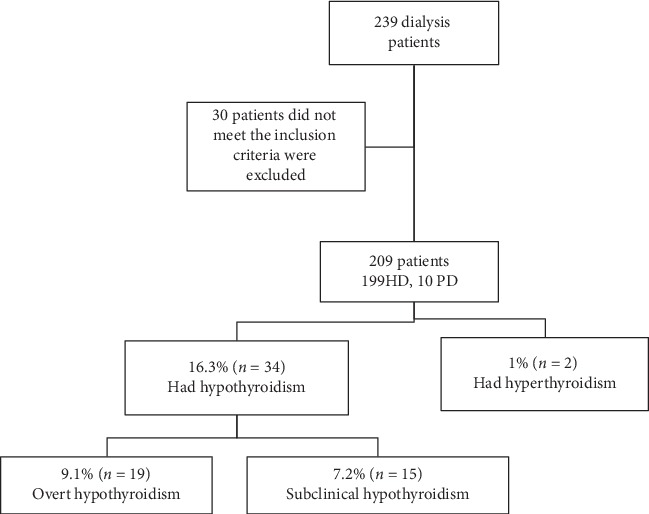
Algorithm of study creation and results. HD, hemodialysis; PD, peritoneal dialysis.

This cross-sectional study was conducted in the dialysis unit of An-Najah National University Hospital (NNUH); the main referral and teaching hospital in Palestine. The dialysis unit is one of the largest units in West Bank with a capacity of 332 dialysis patients monthly. The study was conducted over a period of two months, from 19 September to 19 November 2019. During the study period, there were 332 patients receiving maintenance dialysis in the NNUH. Exclusion criteria included patients known to have thyroid disorders, taking thyroid hormones, had thyroidotomy, and had a family history of thyroid disorders, patients on medication known to affect the thyroid gland (amiodarone, glucocorticoids >50 mg, phenytoin, or lithium), age under 18 years, and patients who recently underwent contrast imaging. Sample size calculation, considering the total population of hemodialysis patients in Palestine is 2071 with 95% confidence interval and expected prevalence of 20%, equals 220 patients. We interviewed and invited 239 patients. After excluding 30 patients, a total of 209 patients receiving maintenance dialysis (199 under hemodialysis and 10 under ambulatory peritoneal dialysis) were included in this study (Figure 1). Hemodialysis patients were dialyzed in classical dialysis (not hemofiltration or hemodiafiltration), using high-flux filters. Majority of the participants received 3.5 hours, on average, of hemodialysis three times a week.

Demographic data (age, gender, and weight), clinical history (comorbidities like diabetes and cardiovascular diseases, smoking history, and medications), and dialysis-related data (duration of dialysis, frequency, and type of dialysis) were collected for all participants.

Approval of the study was obtained, in advance, from the Institutional Review Board (IRB) of An-Najah National University (ANU). Patients were invited to participate in the study voluntarily after they were explained the nature and purpose of the study. All participants provided written informed consent, and their privacy and confidentiality were ensured.

### 2.2. Measurements

Blood samples were taken from all patients through an arterio-venous fistula (or from a venous catheter in those who had it), in the fasting state, before midweek session and heparin administration in the morning. Thyroid hormone testing was done at the laboratory department of NNUH using an immunoassay analyzer; Cobas® e601 analyzer (USA production). Reference ranges for TSH and FT4 were 0.27–4.2 *μ*IU/mL and 12–22 pmol/L, respectively. TSH testing was done for all patients, and FT4 was done to those who had high or low TSH to categorize whether subclinical or overt type.

Overt and subclinical hypothyroidism definition was adopted based on the American Thyroid Association guidelines 2012 and the European Thyroid Association guidelines ATA/AACE 2013 [17, 18]. Hypothyroidism was defined as TSH level >4.2 *μ*IU/mL + normal or low FT4 and categorized based on FT4 as overt hypothyroidism, if low FT4 (<12 pmol/L) and high TSH, and subclinical hypothyroidism, if high TSH and normal FT4. Overt hyperthyroidism was defined as TSH lower than 0.22 *μ*IU/mL and FT4 higher than 22 *μ*IU/mL, and subclinical hyperthyroidism was low TSH and normal FT4.

In Palestine, each dialysis patient undergoes a routine monthly biochemistry profile that includes blood urea nitrogen; serum levels of albumin, sodium, potassium, bicarbonate, creatinine, parathyroid hormone, calcium, phosphate, ferritin, and hemoglobin. Data for the foregoing variables were therefore available for the same month when the thyroid function tests were done.

### 2.3. Statistical Analyses

The data generated from the study were coded and entered into Microsoft Excel and analyzed using IBM SPSS statistics software (version 21.00). Data were expressed as mean ± standard deviation (SD), median and interquartile range (IQR), and percentiles. The significance between variables was tested for the numerical variables using the independent *t*-test or Mann–Whitney *U* test and for categorical variables using chi-square, as appropriate depending on normality testing. *P* value was considered significant if below 0.05.

## 3. Results

Table 1 summarizes the demographic and clinical characteristics of the participants. The mean age of dialysis patients was 57.6 ± 14.5 years, and 64% were males. The median (IQR) of time since the beginning of dialysis was 36 (54) months. Diabetic nephropathy and hypertensive nephropathy were the most common causes of renal failure in the studied sample, 34.5% and 17.2%, respectively, and 7.7% of them had both. Other causes were glomerulonephritis 10%, analgesic nephropathy 5%, polycystic kidney disease 2%, and obstructive uropathy 3%, and 13.9% had no known cause. The majority of patients were on calcium and vitamin D medications.

**Figure 2:**
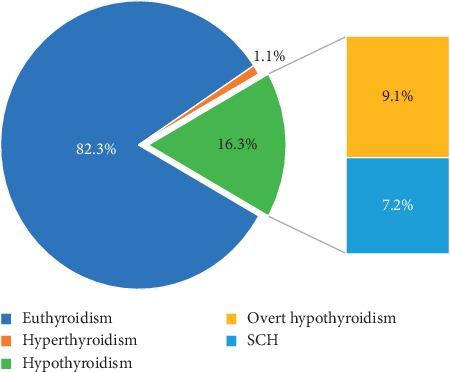
Distribution of thyroid function disorder among patients on maintenance dialysis (*n* = 209).

Among the 209 patients enrolled in this study, we found 34 patients (16.3% (95% CI = 11.29%–21.3%)) with hypothyroidism. The median (IQR) for TSH level was 2.13 (2.25). Of them, 55.9% (*n* = 19) were found to have overt hypothyroidism; which constitutes 9.1% of the total sample, and 7.2% were subclinical hypothyroidism (Figure 2). Only 2 patients had subclinical hyperthyroidism (1%), and none had overt hyperthyroidism.

We compared baseline characteristics between euthyroid with hypothyroidism patients (Table 1) and euthyroid with overt and SCH patients (Table 2). No significant difference in age, gender, duration of dialysis, weight, or comorbidities (hypertension and diabetes) was found when compared to euthyroid. As for laboratory variables, creatinine was significantly lower in hypothyroid and overt hypothyroid patients compared to euthyroid patients (*P*=0.018) and (*P*=0.011) respectively, and we also detected a significant difference between SCH and euthyroid in bicarbonate level (*P*=0.077).

Hypothyroidism symptoms were observed to be common not only in hypothyroid patients but also in euthyroid patients with an almost similar distribution. For example, a feeling of being fatigued was reported by 33.9% of euthyroid patients and 38.2% of hypothyroid patients (Table 3).

## 4. Discussion

The kidney has an important role in the metabolism of thyroid hormones, and patients on maintenance dialysis have a higher risk than healthy people for having thyroid abnormalities, but the exact prevalence varies between studies and countries. In this study, the prevalence of hypothyroidism was 16.3% (95% CI = 11.29%–21.3%), in which 9.6% were overt hypothyroidism and 6.7% were subclinical hypothyroidism. The prevalence of hypothyroidism reported in this study is higher compared to the prevalence of hypothyroidism (as high TSH) among general population in Palestine, which was reported by Amro et al. as 5.3% [19]. Our results are comparable to those in other regions around the world. For example, Kutlay et al. reported in their study, in Turkey, the prevalence of hypothyroidism (high TSH) and SCH was 23.1% and 19.7%, respectively [5]. They had a similar prevalence rate of hypothyroidism, but the rate of subclinical hypothyroidism was much higher than in our study. In Brazil, Da Costa et al. showed higher prevalence rates of both hypothyroidism and SCH, in which their frequency of hypothyroidism was 27.12% and SCH was 21.82% [3]. On the other hand, we had a higher prevalence of overt hypothyroidism compared to both abovementioned studies, considering that overt hypothyroidism is the difference between hypothyroidism and SCH prevalence. Similarly, in Shantha et al.'s study [20], in 137 hemodialysis patients tested for TSH and FT4, SCH prevalence was 24.8%, also higher than our findings. Although the previously mentioned studies were cross-sectional studies, like our study, results from prospective studies like in Jusufovic et al.'s study are not very different, in which their incidence of overt hypothyroidism was 5% and SCH was 17.5% [7].

The strength of our study compared to the abovementioned ones is that our sample size is larger. Nevertheless, all agreed that dialysis patients had a high prevalence of hypothyroidism, and even confirmed in a larger-scale study of 8840 hemodialysis patients based on baseline TSH, they observed that 22% of them had hypothyroidism, but they did not test FT4, so they had no results about overt and subclinical hypothyroidism [13]. Thus, one can conclude that the prevalence of hypothyroidism defined as high TSH is similar in different population but varies in terms of its category (SCH and overt hypothyroidism), and this was observed in our population in which we had the lowest number of subclinical hypothyroidism and the highest number of overt hypothyroidism compared to many studies. This difference has a very important inference, as the latest recommendations for treating hypothyroidism are against treating SCH [21], and this lead clinicians in other population not to have any intervention for dialysis patients as most are SCH, but this is not the case in our population in which the majority are indeed overt hypothyroidism, which highlights the importance of screening and interventional programs for this population.

An important point to consider in this regard is that studies had different definitions for hypothyroidism, and this arises from the arguments about the accepted TSH level in dialysis, as some authors propose that levels between 5 and 20 IU/ml are considered normal in dialysis patients and so no treatment is required, though in studies that assessed mortality found that TSH even in high normal range (3–5 *μ*IU/ml) was associated with a higher risk of mortality [13]. Accordingly, in this study, we considered TSH higher than 4.2 *μ*IU/ml as abnormal. However, Kutlay et al. considered a level of TSH >5.5 *μ*IU/ml in their definition of hypothyroidism, and Jusufovic et al.' definition involves a TSH level higher than 10 *μ*IU/ml. Consequently, this could be a reason for the noted variations in the prevalence of hypothyroidism in the literature in addition to geographic differences.

Hyperthyroidism is less concerning in this population as the majority of studies show no significant difference compared to healthy controls [6, 7, 22]. In our study, the prevalence of hyperthyroidism was 1%, similar to the results by Kutlay et al. [5], while Jusufovic et al. [7] reported a higher prevalence of hyperthyroidism in their study (2.5%), but still not significant compared to controls.

Comparison between peritoneal dialysis and hemodialysis patients yields no difference between the prevalence of thyroid disorders. Though, due to the small sample of peritoneal dialysis patients, we could not investigate this comparison. Additionally, previous studies confirmed no significant difference between TSH level in hemodialysis and peritoneal dialysis, and no significant difference existed between characteristics of patients under hemodialysis and those under peritoneal dialysis [11, 22]. In other words, our results were considered for dialysis patients in general regardless of the type of dialysis.

The controversies over the patients' characteristics that may be associated with thyroid disorders still exist. Some studies showed that thyroid abnormalities are higher in females [12], while others, as in this study, were insignificant [3, 11, 20]. Though we had more males (60% of our sample), they accounted only for 47% of overt hypothyroid patients. Higher prevalence of hypothyroidism was noted with a longer duration of dialysis [12], and no significant difference was found in our study as many others [3, 5, 7]. We also did not demonstrate any significant difference when comparing the hypothyroid state based on age, comorbidities, and weight. As for laboratory tests, we found a significant relation with serum creatinine level, in which hypothyroidism was associated with a lower level. Other studies found that low creatinine level is associated with a higher risk of death in dialysis patients [23], and this may be one of the reasons why hypothyroidism increases mortality and morbidity, but more studies need to be conducted to investigate this theory. Shantha et al. reported that albumin was significantly lower in SCH [20], but in our study, we found no significant association.

Bicarbonate was significantly different only when comparing SCH to euthyroid patients, which was lower (acidosis state) in SCH though it can be attributed to metabolic acidosis effect on thyroid faction, and this would be an acceptable explanation if the difference significance was with overt hypothyroidism, perhaps further studies can illustrate this link.

Hypothyroidism symptoms observed to be common in both hypothyroid and euthyroid patients, as we observed that common symptoms like fatigue, cold intolerance, and depression are commonly reported by dialysis patients even in euthyroid ones. This makes it difficult to rely on symptoms to indicate the presence of hypothyroidism, and this again necessitates a screening program as many authors suggested.

Limitation of this study is that it was a cross-sectional study; therefore, cause-effect relationships cannot be assessed. Moreover, we did not estimate the prevalence of low FT4 and FT3 since it was, especially low FT3, associated with mortality and worse prognosis [14]; since our focus was on hypothyroidism and hyperthyroidism, FT3 is not included in the definition, and TSH is the most reliable indicator of thyroid function [9]. Furthermore, antithyroid antibodies were not tested in our study, which may help to find the etiology whether a primary thyroid abnormality or due to renal impairment. Also, we cannot assure the patient's thyroid state before dialysis, as we relied only on the patient's knowledge of their state. Finally, we did not have a comparison group of healthy controls to study the differences between them, but we compared with other studies in our population and different ones.

## 5. Conclusion

Dialysis patients had a high prevalence of hypothyroidism, both SCH and overt hypothyroidism. Hypothyroidism symptoms are common in dialysis patients even in euthyroid. This makes the diagnosis of hypothyroidism difficult, and it can be easily missed in the ESRD population because of the overlap between ESRD symptoms and hypothyroidism.

Early diagnosis and proper management of hypothyroidism prevents deterioration of patient's conditions and prolong survival. Taking into consideration the high prevalence rate, physicians should pay attention to this condition and screen routinely for thyroid function disorders in the dialysis population. Further studies to assess the efficacy of thyroid hormone supplement and its long-term effects in decreasing mortality and morbidity are recommended.

## Figures and Tables

**Table 1:** Background and clinical characteristics of the study participants and its relation to hypothyroidism (*n* = 209).

Characteristic	Total	Euthyroid	Hypothyroidism (high TSH)	*P* value
Age in years^*∗*1^	60 (21)	59 (21)	60 (15)	0.599

Gender %(*n*)				
Male	64.1% (134)	66.1% (113)	52.9% (18)	0.172

Duration of dialysis, (mo)^*∗*1^	36 (54)	36 (54)	36 (63)	0.597

Weight, kg^*∗*1^	74 (24)	74.5 (23.3)	23.3 (32.4)	0.611

Comorbidities, % (*n*)				
Diabetes	56.5% (118)	55% (77)	61.8% (34)	0.571
Hypertension	80.4% (168)	80.1% (137)	82.4% (28)	0.488
Cardiovascular disease	52.2% (109)	52.5% (88)	55.9% (19)	0.709
Heart failure	27.3% (57)	34.4% (44)	43.3% (13)	0.401
IHD	32.1% (67)	39.6% (55)	37% (10)	1
Smoker	25.4% (53)	28.7% (49)	8.8% (3)	0.017

Laboratory tests^*∗*^				
Albumin, serum (g/dL)	3.83 (0.46)	3.83 (44)	3.84 (61)	0.654
Creatinine, serum (mg/dL)	8.19 (3.32)	8.35 (3.55)	7.12 (3.53)	0.018
Ferritin (ng/ml)	545.4 (397)	538.8 (413.80)	642.25 (369)	0.384
Hemoglobin (g/dL)	10.94 (1.66)	11 (1.58)	10.47 (1.86)	0.109
Ca, serum^#^ (mg/dL)	9.16 (0.88)	9.18 (0.92)	9.17 (0.77)	0.607
Bicarbonate (mEq/L)	20.3 (3.5)	20.5 (3.4)	19.4 (4.3)	0.077
P, serum (mg/dL)	4.44 (1.89)	4.44 (1.88)	4.49 (2.56)	0.843
PTH, (pg/mL)	309.9 (353.8)	304.4 (379.87)	340.65 (222.4)	0.543
BUN, serum (mg/dL)	52.3 (15.0)	52.05 (13.78)	55.3 (19.13)	0.939
Potassium (mEq/L)	5.06 (1.05)	5.06 (1.07)	5.14 (84)	0.750
Sodium (mEq/L)	138 (4.0)	138 (4)	138.5 (3)	0.380
Chloride (mg/dl)	97.80 (4.5)	97.4 (4.74)	98.1 (3.15)	0.307

^*∗*1^Median (interquartile range). ^#^Corrected for albumin; P, phosphate; PTH, parathyroid hormone; Ca, calcium; BUN, blood urea nitrogen; IHD, ischemic heart disease.

**Table 2:** Association of patients' characteristics with overt hypothyroidism and with SCH.

Characteristic	Euthyroid	Overt hypothyroidism	*P* value	SCH	*P* value
Age in years ^*∗*^	59 (21)	62 (16)	0.498	58 (14)	0.879

Gender % (*n*)					
Male	66.1% (113)	47.4% (9)	0.131	60% (9)	0.778

Duration of dialysis, (mo)^*∗*^	36 (54)	30 (39)	0.275	48 (72.0)	0.741

Weight, kg^*∗*^	74.5 (23.3)	76 (35.5)	0.785	67 (33.0)	0.622

Comorbidities, %					
Diabetes	55% (77)	57.9% (11)	1	66.7% (10)	0.429
Hypertension	80.1 (137)	89.5% (17)	0.537	72.3% (11)	0.513
Cardiovascular disease	51.5% (88)	57.9% (11)	0.636	53.3% (8)	1
Heart failure	34.4% (44)	47.1% (8)	0.42	38.5% (5)	0.767
IHD	39.6% (55)	35.7% (5)	1	38.5% (5)	1
Smoker	28.7% (49)	10.5% (2)	0.107	6.7% (1)	0.074

Laboratory tests^*∗*^					
Albumin, serum (g/dL)	3.83 (44)	3.81 (60)	0.332	3.9 (.65)	0.676
Creatinine, serum (mg/dL)	8.35 (3.55)	6.84 (3.37)	0.011	374.1 (3.72)	0.388
Ferritin (ng/ml)	538.8 (413.8)	522.2 (452.7)	0.457	650.8 (374.1)	0.596
Hemoglobin (g/dL)	11 (1.6)	10.4 (1.8)	0.123	10.8 (1.67)	0.434
Ca, serum^#^ (mg/dL)	9.18 (0.92)	9.52 (0.69)	0.292	9.14 (1.06)	0.7
Bicarbonate, (mEq/L)	20.5 (3.4)	21.5 (4.6)	0.878	18.4 (3.3)	0.009
P, serum (mg/dL)	4.44 (1.88)	4.48 (2.81)	0.972	5 (2.34)	0.785
PTH, (pg/mL)	304.4 (379.87)	356.2 (222.3)	.588	324.7 (246.8)	0.730
BUN, serum (mg/dL)	52.05 (13.78)	48.7 (17)	0.408	58 (25.2)	0.289
Potassium (mEq/L)	5.06 (1.07)	4.99 (73)	0.518	5.29 (.83)	0.216
Sodium (mEq/L)	138 (4)	139 (3.00)	0.256	138 (2)	0.926
Chloride (mg/dl)	97.4 (4.74)	98.3 (3.30)	0.459	98 (3.10)	0.440

^*∗*^Median (interquartile range). ^#^Corrected for albumin; P, phosphate; PTH, parathyroid hormone; Ca, calcium; BUN, blood urea nitrogen; SCH, subclinical hypothyroidism; IHD, ischemic heart disease.

**Table 3:** Frequency of hypothyroid symptoms in euthyroid and hypothyroid patients.

Symptoms	Euthyroid (%)	Overt hypothyroidism (%)	Subclinical hypothyroidism (%)	*P* value
Fatigue	33.9	42.1	33.3	0.802
Cold intolerance	26.3	47.4	46.7	0.06
Weight gain	8.8	15.8	13.3	0.560
Inability to concentrate	11.7	15.8	13.3	0.693
Depression^*∗*^	12.9	10.5	15.4	0.301
Skin dryness	12.3	12.3	20	0.158

^*∗*^Patients' perceived feeling.

## Data Availability

The data used to support the findings of this study are available from the corresponding author upon request.
